# Responsiveness of the PROMIS-10 global compared to the neck disability index in patients undergoing 1 and 2 level anterior cervical discectomy and fusion

**DOI:** 10.1016/j.xnsj.2025.100780

**Published:** 2025-08-05

**Authors:** Matthew J. Solomito, Heeren Makanji

**Affiliations:** a Orthopedic Research Department, 31 Seymour St. Hartford HealthCare Bone and Joint Institute, Hartford, CT, 06106 United States; b Spine Surgery, Orthopedic Associates of Hartford, 31 Seymour St. Hartford, Hartford, CT, 06106, United States

**Keywords:** ACDF, Cervical spine, NDI, Outcomes, Patient reported outcomes, PROMIS, Responsiveness

## Abstract

**Background:**

The reliance on patient reported outcomes (PROs) has substantially increased not only to augment current metrics of clinical success, but to capture the patient’s perspective on the benefit of their treatment. As more PROs become utilized, the time and cost of longitudinal data collection and survey fatigue must be tempered with the benefit of the data collected. Therefore, this study sought to assess the responsiveness of the Neck Disability Index (NDI) compared to the PROMIS-10 Global Health Survey physical function T-score (PFT) and mental health T-score (MHT).

**Methods:**

A total of 264 patients that had undergone a single or two level anterior cervical discectomy and fusion (ACDF) between June 2021 and January 2024 were included. All patients completed their preoperative, 3-, and 12-month postoperative PRO assessments. A responsiveness analysis was performed and included: floor and ceiling effects, correlations among the PRO scores, and effect size indices (ESI) calculations.

**Results:**

There were no floor or ceiling effects for the NDI and only 5.8% of the study cohort reached the floor or ceiling for the PROMIS-10 scores. The PROMIS T-scores showed weak to moderate correlations to the NDI, with the PFT having stronger correlations than the mental health T-score (MHT). The ESI demonstrated that the NDI was the most responsive tool with a maximum ESI of 0.98.

**Conclusions:**

The PROMIS-10 is a responsive and valid tool that provides insight into both the general physical function and mental health of a patient; however, it does not display the same discretionary ability to detect small changes in neck function that the NDI demonstrated. Therefore, the PROMIS-10 may be useful to provide preoperative assessment for patients undergoing ACDF but longitudinal evaluation to assess the outcomes of this surgery may be best left to the NDI.

## Introduction

Patient reported outcomes (PROs) were developed as a means to understand and standardize the collection of a patient’s perspective on the benefit and efficacy of their care [1]. In the era of value-based care, the reliance on PROs has substantially increased not only to augment current metrics of clinical success [[2], [3], [4]], but to also to help combat the rising costs associated with the United States Healthcare System [[5], [6], [7], [8]]. Many providers use these data as a means of managing patient expectations and to facilitate shared decision-making. Furthermore, recent studies have demonstrated the utility of preoperative PRO data to predict postoperative outcomes [[9], [10], [11]]. As PROs use continues to grow the benefits of collecting PRO data must be tempered with the growing concerns regarding survey fatigue and assessed in conjunction with streamlining PRO assessment without compromising predictive abilities.

The NDI was first developed in 1991, and was designed to specifically assess disability and pain in patients with neck pathology [12]. Since its creation, the NDI has proven to be a responsive, consistent, and reliable PRO [[13], [14], [15]]. The NDI has become the most widely used neck and cervical spine assessment tool due to the extensive scientific rigor assessing the NDI’s utility in clinical settings [12]. However, more recently, quality of life PROs (eg, SF-36 and EQ-5D) have also become more widespread in their utilization in both clinical and research settings. Most recently, the Patient-Reported Outcomes Measure Information System Global Health Survey (PROMIS-10), was developed by the National Institute of Health, and has become increasingly adopted in the setting of orthopedic surgery. The PROMIS-10 assesses both the patient’s physical function and mental health through two subdomains within its 10 question construct. Unlike the NDI, the PROMIS-10 is not a disease specific measure, but rather an assessment of global health [[16], [17], [18]], and recent studies have shown that the PROMIS-10 provides similar results to other quality of life surveys in a more efficient format [[19], [20], [21], [22], [23], [24], [25]]. Additionally, the PROMiS-10 utilizes a unique scoring system that allows for direct comparison of a patient’s overall health to that of the United States population using T-score mapping.

Recent studies have begun to examine the utility of these quality-of-life surveys within the context of more common disease specific legacy PROs (ie, the Oswestry Disability Index (ODI) and Knee Injury and Osteoarthritis Outcomes Score for joint Replacement) in an effort to streamline and reduce PRO collection for patients [17,26,27]. However, while the current body of literature surrounding the responsiveness of the PROMIS-10 compared to legacy PROs continues to grow, there has been a paucity of investigations evaluating the responsiveness of the PROMIS-10 physical function T-score (PFT) and mental health T-score (MHT) with the NDI. Therefore, the purpose of this study was to evaluate the responsiveness of the PROMIS-10 Global’s mental health and physical function subdomain scores compared to the Neck Disability Index to determine if these PROs provided a similar evaluation of patient perceived function and outcomes.

## Methods

This retrospective study was approved by our center’s Institutional Review Board. Patient records were included if the patients were: between the ages of 18 and 89 years old, underwent a single or two level, elective anterior cervical discectomy and fusion (ACDF) between June 2021 and January 2024, and completed their preoperative NDI and PROMIS-10 as well as the NDI and PROMIS-10 at 3- and 12-months post fusion using Force Therapeutics (FORCE Therapeutics, New York, NY), an online patient engagement platform used to capture patient reported outcomes. Patient records were excluded if patients: underwent a fusion of three or more levels, had a staged procedure, had a history of dementia, cognitive deficit, illicit drug use, or known opioid dependence. Patient records were also excluded if the patient had undergone another surgical procedure within 13 months of their index procedure, or if the indication for fusion was trauma or pathological condition (ie, cancer).

To determine the responsiveness of the PROMIS-10 and the NDI three analyses were performed. First the PRO constructs were assessed for floor and ceiling effects to determine the percentage of patients that reached either the maximum, ceiling, or minimum, floor, scores. Determination of the floor and ceiling effects provided an indication of how well each of the scales were at identifying patients near the extreme ends of constructs [28]. Second effect size indices (ESI) were calculated for both the NDI and PROMIS 10 as a means of assessing the responsiveness of the constructs at the post fusion assessments [[29], [30], [31], [32]]. Responsiveness evaluations indicate that larger ESI values indicate that the PRO can better detect change between subsequent time points [26]. For the purposes of this study an ESI of 0.2 or less was considered a low effect, an ESI greater than 0.2 but less than 0.8 was a moderate effect, and an ESI of 0.8 or greater was considered a large effect. Finally, a Pearson’s correlation was performed to determine how closely the related the NDI and PROMIS-10 scores were at measuring the same population. The correlation coefficients interpretations were defined as: less than 0.2 no correlation, 0.2 to 0.5 a weak correlation, 0.5 to 0.7 a moderate correlation, 0.7 to 0.9 a strong correlation, and greater than 0.9 as a near perfect correlation. All statistical testing was performed using STATA SE17 (StataCorp, College Station, TX).

## Results

**Fig. 1:**
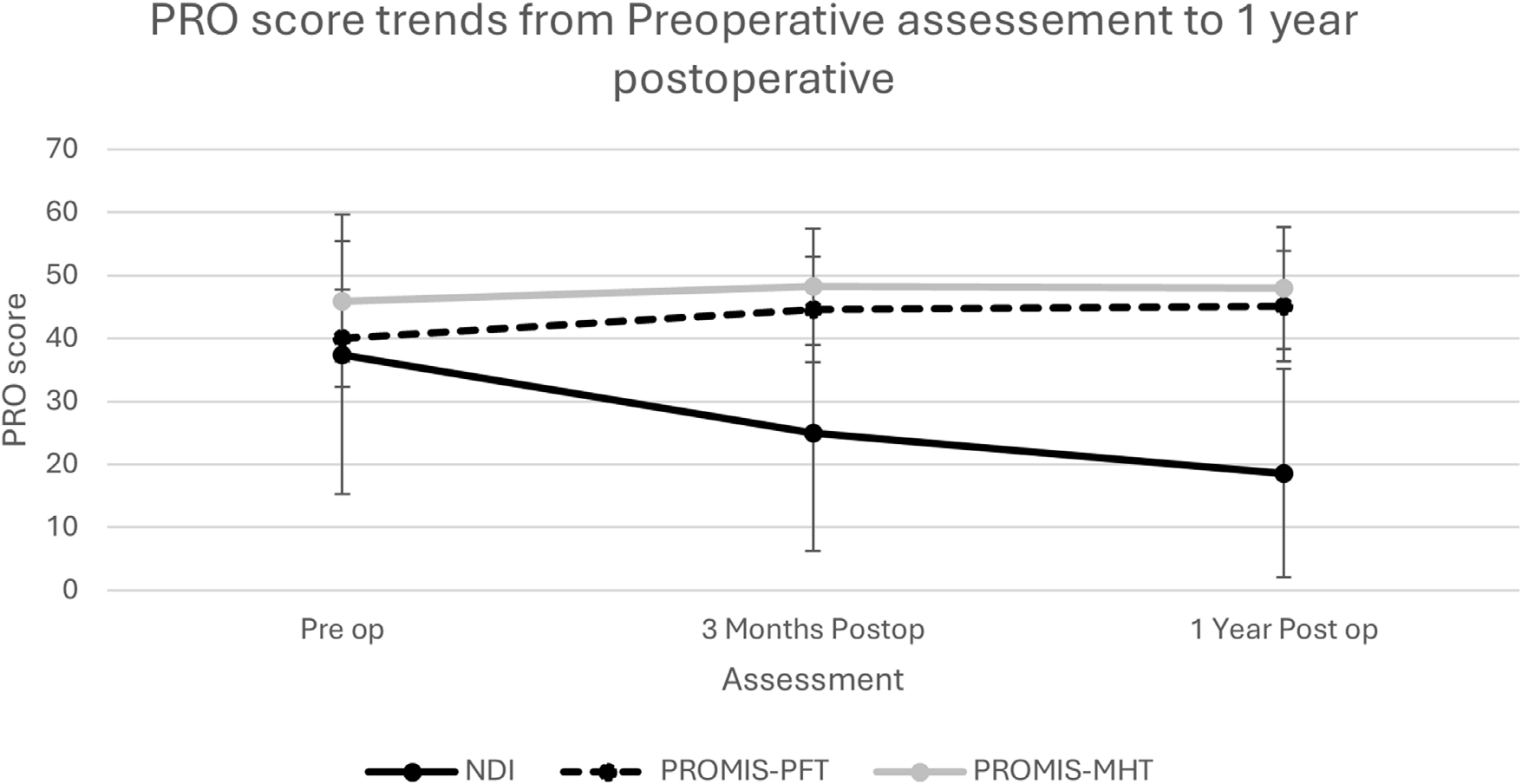
Average patient reported outcome (PRO) score trends and one standard deviation from the initial preoperative assessment through 1-year postoperative follow-up. Solid Black line represents the Neck Disability Index (NDI), the solid grey line represents the PROMIS-10 Mental Health T-score (MHT), and the dotted black line represents the PROMIS-10 Physical Function T-score (PFT).

A total of 264 patients were included in this study (Table 1). The average PROMIS T-scores indicated that preoperatively most patients reported average physical function and mental health. The NDI score that indicated the majority of patients believed their spinal condition led to moderate to severe disability (Fig. 1). All patients showed significant improvement by 3 months postoperatively for the NDI and PFT but showed limited score change for the MHT. The NDI score continued to improve through 1 year after ACDF while the PROMIS showed minimal additional improvements after 3 months (Fig. 1).

**Table 1:** Descriptive parameters of the study cohort.

Parameter	
N	264
Age	59.0±11.4
Height (in)	66.1±4.0
Weight (lb)	193.7±50.9
BMI (kg/m^2^)	30.9±7.0
CCI	2±2
Sex	
Male	107 (40.5%)
Female	157 (59.5%)
Race	
White	221 (84.1%)
African American	19 (7.2%)
Other	24 (8.7%)
Ethnicity	
Not Hispanic	248 (93.9%)
Hispanic	16 (6.1%)
Insurance	
Commercial	8 (3.0%)
Medicare	210 (79.5%)
Medicaid	30 (11.4%)
Other government	16 (6.1%)
Levels	
1	119 (45.1%)
2	145 (54.9%)

There was no floor or ceiling effect for the NDI, since none of the patients within this cohort scored the minimum or maximum value for the NDI at any of the three assessed time points; however, patients did meet both the floor and ceiling of the PROMIS (Table 2). In regard to the PROMIS PFT none of the patients reached the floor at any time point; however, two patients (0.8%) reached the ceiling at 3 months post fusion and 5 patients (1.9%) reached the ceiling at 1 year post fusion. Patients met both the floor and ceiling for the PROMIS-MHT scores. The floor was reached by 3 patients (1.1%) preoperative, no patients at 3 months, and 1 patient (0.4%) at 1 year post fusion. The ceiling for the PROMIS-MHT score was reached by 5 patients (1.9%) preoperatively, 12 patients (4.5%) 3 months postoperatively, and 15 patients (5.7%) by 1 year post fusion.

**Table 2:** Comparison of the Oswestry disability index, PROMIS-10 physical function T-score, and PROMIS-10 mental health T-score from baseline to 1 year post fusion.

	Baseline	3 months postop	12 months postop	p-value
NDI	37.4±22.2	25.0±18.7*	18.6±16.5*	<.001
score range	9–96	3–84	9–90	
PROMIS-MHT	45.9±9.5	48.2±9.2	47.9±9.6	.101
score range	21.2–67.7	25.1–67.7	21.2–67.7	
PROMIS-PFT	40.0±7.6	44.6±8.4	45.1±8.7*	.020
score range	23.5–61.7	23.5–67.7	23.5–67.7	

NDI, neck disability index, MHT, mental health T-score, PFT, physical function T-score.

⁎ Significant difference compared to baseline.

Pearson correlation indicated that there was a weak to moderate correlation between the NDI and PROMIS-PFT scores, and weak correlations between the NDI and PROMIS-MHT scores (Table 3). The responsiveness analysis (Table 4) based on the ESI indicated that the NDI was very responsive with large effect sizes of 0.82 and 0.96 at 3 months and 1 year post fusion. The PROMIS responsiveness indicated moderate responsiveness for the PFT score with ESI values of 0.55 and 0.67 at 3 months and 1 year post fusion. However, the PROMIS MHT scores demonstrated poor responsiveness at both 3 months and 1 year post fusion with ESI values of 0.33 and 0.22 respectively.

**Table 3:** Correlation coefficients between the neck disability index (NDI) and the PROMIS mental health T-score (MHT) and the PROMIS physical function T-score (PFT) from baseline to 12 months post fusion.

	Baseline	3 months	12 months
NDI to PROMIS MHT	−0.35	−0.41	−0.45
NDI to PROMIS PFT	−0.48	−0.54	−0.58

**Table 4:** Effect Size indices describing the responsiveness of the neck disability index (NDI), the PROMIS mental health T-score (MHT), and the PROMIS physical function T-score (PFT) assessed between baseline and each of the three follow-up assessments.

	3 months	12 months
NDI	0.82	0.96
PROMIS PFT	0.55	0.67
PROMIS MHT	0.33	0.22

## Discussion

Patient reported outcomes have become a standard metric to assess orthopedic outcomes in both a clinical and research setting. Although the NDI has been seen as the gold standard to assess cervical spine pathology and success of treatment modalities, the PROMIS-10 Global quality of life questionnaire has begun to gain widespread adoption throughout orthopedic subspecialties due to its short and concise format, and easy-to-interpret scoring system. As a result, patients are often required to complete both the NDI and PROMIS-10 as part of their routine clinical assessment and follow-up. Although both surveys are relatively short constructs requiring only 10 to 15 minutes of time for a patient to complete, in busy practices this time could affect efficiency; additionally, some patients quickly tire of completing longitudinal forms, and as a result compliance and follow-up assessments sharply decline. Therefore, finding a way to optimize the longitudinal collection of PROs in a way to maximize actionable data while reducing survey burden and cost of data collection is of great importance. Therefore studies, such as this, that are designed to objectively evaluate the utility of questionnaires currently used in clinical practice are essential to determine if the surveys provide substantial clinical benefit. The results of this study demonstrated that patient reported function improves greatly from baseline to 3 months post ACDF; however, the patients mental wellbeing remains relatively constant over the entire assessment period.

Overall, the results of this study indicated that while the PROMIS-10 is a valid and responsive measure it may not be the best PRO to fully assess the current function of a patient suffering from cervical spine pathology, nor is it the best tool to fully assess a patient’s functional recovery following an ACDF. This conclusion was based on the relatively low ESI values noted for both the PROMIS-MHT and PROMIS-PFT scores when compared to the substantially greater ESI values for the NDI. This is further supported by the fact that the NDI scores continued to improve from the preoperative assessment through the 1 year post operative assessment while the PROMIS scores show limited improvement between the preoperative and 3-month assessments and remain nearly stagnant through the 1-year post ACDF assessment. This is one of the first studies to compare the PROMIS-10 PFT and MHT scores to the NDI scores using an ESI analysis and thus comparative results within literature are lacking. However, a recent study assessing the ODI and PROMIS-10 using and ESI analysis demonstrated that, in contrast to this study, the PFT was far more responsive in a lumbar spine population that seen in this study and was comparable with the ODI [27]. However, the results indicating that the MHT is not a responsive measure in this population is consistent with previous works that indicated the MHT shows limited variation from preoperative to postoperative states [27,33,34].

The low floor and ceiling effects for both the PROMIS-10 PFT, MHT, and NDI demonstrated that all of the constructs provide a discriminatory power for patient responses at both ends of the scales. This suggests that the constructs are accurate and limit the potential for response bias [19,28]. The MHT did have more patients, more patients reach both the floor and ceiling effect than both the PFT and NDI; however, the number of patients reaching these values was relatively low. It is also important to note that given that the PROMIS-10 T-score mapping places patients into categories (eg, below average, average, above average etc.) reaching an end point would be less concerning than at a value closer to a transition point between T-score groups, and therefore, may not be a detractor for this PRO.

The results showed weak to moderate correlations between the PROMIS-PFT and NDI scores with correlation coefficients ranging between 0.48 and 0.58 from the preoperative assessment through the 1-year postoperative assessment. These findings are quite different than previous studies that looked at the correlations between the PFT and NDI in patients with cervical spine pathology, and demonstrated moderate to strong correlations between the physical function domain and NDI. However, in these studies the cervical spine pathology and treatment was not as homogeneous as this study assessing numerous fusion levels, ACDFs, posterior fusion approaches, and isolated discectomies, which could lead to variations in the results when compared to this current study. Additionally, previous studies conducted with a total joint arthroplasty population and lumbar spine population have indicated significant correlation between the PFT and the legacy PRO scores used within those subspecialties [17,26,27]. However, it is important to note that the PROMIS-10 questions were designed for general health and function, and some of the questions are worded emphasizing lower extremity function (ie, walking, climbing stairs, and moving a chair); while this is not meant as a criticism to the PROMIS-10, this may lead to patients who are experiencing upper extremity and neck impairment to answer this question differently than those with lower extremity involvement. Therefore, this may explain the reduced correlation coefficients seen in this study in comparison to the studies that focused on populations that suffer lumbar spine pathology or end stage hip or knee osteoarthritis. Additional investigation is necessary to confirm this concept.

This study is not without limitations. The study cohort was skewed towards a Caucasian Medicare population, which makes the results of this study less generalizable to the general population, or a population with greater diversity. Furthermore, inclusion criteria for this study required that patients complete their PROMIS-10 and NDI at the preoperative, 3 months postoperative, and 1 year postoperative time points, and therefore may have led to unintended selection bias, given that the institutional completion rate for longitudinal PRO completion is around 64%.

## Conclusion

The results of this study demonstrated that the NDI is the most responsive construct assessed within this study to detect functional changes following elective 1 to 2 level ACDFs, and this study supports its continued use as the primary PRO for assessing patients undergoing ACDFs. Although the PROMIS-10 is a responsive and valid tool that provides insight into both the general physical function and mental health of a patient, it does not display the same discretionary ability to detect small changes in neck function that the NDI demonstrated. That being said, the PROMIS-10 should not be completely discounted in this population given that the weak to moderate correlation between the NDI and PROMIS suggests that the PROMIS is providing data different than what is captured by the NDI. Therefore, the PROMIS-10 may be useful to provide preoperative assessment for patients undergoing ACDF but longitudinal evaluation to assess the outcomes of this surgery may be best left to the NDI.

## Declaration of competing interest

The authors declare that they have no known competing financial interests or personal relationships that could have appeared to influence the work reported in this paper.
